# Clinical Information and Prognosis of High-risk Luminal Breast Cancer Subjects Eligible for the MonarhE Study

**DOI:** 10.31662/jmaj.2024-0243

**Published:** 2025-02-14

**Authors:** Mio Adachi, Toshiyuki Ishiba, Sakiko Maruya, Kumiko Hayashi, Yuichi Kumaki, Goshi Oda, Tomoyuki Aruga

**Affiliations:** 1 Department of Breast surgery, Institute of Science Tokyo, Tokyo, Japan; 2 Department of Surgery (Breast), Tokyo Metropolitan Cancer and Infectious Diseases Center Komagome Hospital, Tokyo, Japan

**Keywords:** luminal breast cancer, MonarchE, adjuvant therapy

## Abstract

**Introduction:**

Luminal breast cancer is the most common breast cancer subtype. Although its prognosis can be good, this type of breast cancer is characterized by a high incidence of late recurrence. However, to the best of our knowledge, there are no publications showing prognostic value regarding the invasive-disease-free survival (IDFS) and distant relapse-free survival in this group in clinical practice. Therefore, this study examined the clinical data and prognosis of patients participating in the MonarchE trial.

**Methods:**

This study included patients who underwent surgery at Tokyo Metropolitan Komagome Hospital and whose corresponding prognosis to the Monarch E trial could be followed up.

**Results:**

The total number of participants was 152, of whom 104 (68%) were treated with chemotherapy. Seventy-five patients (49%) were postmenopausal. The IDFS after 5 years was 85.0%. Although IDFS did not differ in terms of the menstrual status, premenopausal patients tended to receive a higher proportion of tamoxifen, and there was a greater number of patients treated with chemotherapy. However, neither chemotherapy nor menstrual statuses were found to affect the IDFS incidence.

**Conclusions:**

Real clinical data applicable to the MonarchE study were examined. Our univariate analysis revealed that there were no factors affecting IDFS.

## Introduction

There are an estimated 220 million patients with breast cancer worldwide ^(1), (2)^. In 2020, a total of 68,000 people passed away from breast cancer, which makes it the fifth leading cause of death globally ^(2), (3)^.

Over 70% of all newly diagnosed breast cancers are luminal breast cancer subtypes (estrogen-receptor positive/human epidermal growth factor receptor 2 [HER2] negative) ^(4), (5)^. While patients with triple-negative and HER2-positive breast cancer tend to have early recurrences, luminal breast cancer is known to recur even more than 10 years after surgery, and late recurrences are more common than for the other two types of breast cancer ^(6)^. Perioperative chemotherapy is entirely based on patient age, tumor size, histologic grade, lymph node metastases, hormone receptor status, and HER2 status ^(7), (8)^, and it has contributed to the decrease in breast cancer mortality recently ^(9)^. Although patients with luminal breast cancer respond well to endocrine therapy and the prognosis is good, this subtype still poses one of the greatest problems associated with poor biological malignancy and prognosis ^(7)^. Depending on clinical and pathological findings, current guidelines recommend the consideration of chemotherapy, hormonal agents, and radiotherapy in the treatment of luminal breast cancer, and the American Society of Clinical Oncology (ASCO) guidelines recommend 10-year endocrine therapy for node-positive breast cancer ^(10), (11)^. Most luminal breast cancers do not recur with standard treatment. However, 20% recur 10 years after surgical resection, resulting in incurable breast cancer ^(6)^. In particularly, the recurrence rate 20 years after surgical resection of breast cancer with a large number of lymph node metastases exceeds 50%; therefore, the biological prognosis of luminal breast cancer is poor ^(12)^. Most cases of recurrences are distant, and postoperative adjuvant therapy is a necessary treatment in patients with high-risk luminal breast cancer ^(13)^.

In addition to conventional treatments such as hormone therapy and chemotherapy, several recent clinical trials have added various drugs to the systemic treatment of luminal breast cancer ^(14), (15), (16), (17)^. For instance, the OlympiA trial showed that administering olaparib postoperatively for 1 year to patients with HER2-negative breast cancer and *BRCA* pathogenic variant improved the invasive-disease-free survival (IDFS) and overall survival (OS) ^(14), (18)^. In the POTENT trial, 1 year of postoperative S-1 therapy in high-risk patients with luminal breast cancer was associated with IDFS ^(15)^. In the PALLAS trial, Mayer *et al.* investigated whether the combination of palbociclib and hormones as a postoperative adjuvant therapy could improve IDFS in hormone-positive, HER2-negative, Stage II and III breast cancer patients ^(16), (19)^. However, their results indicate that there is no additive effect of palbociclib compared with hormone therapy. The NATALEE trial showed that the addition of ribociclib to adjuvant therapy improved IDFS in Stage II and III luminal breast cancer ^(17)^. In the MonarchE trial, which included patients with luminal breast cancer and ≥4axillary lymph node metastases, or 1-3 axillary lymph node metastases, invasion diameter ≥5 cm, histological grade (HG) 3, and Ki67 ≥ 20%, the addition of abemaciclib to postoperative endocrine therapy for 2 years was reported to improve IDFS or distant-relapse-free survival (DRFS) compared to endocrine therapy alone ^(20), (21), (22)^. As ribociclib is unavailable in Japan, there are three types of adjuvant therapy for high-risk luminal breast cancer that can be used in addition to hormone therapy: abemaciclib, olaparib, and S-1.

In general, there is no standard definition of high-risk disease for luminal breast cancer. Although risk factors such as tumor diameter, number of lymph node metastases, HG, and Ki67 help to make a comprehensive judgment of the disease, there are no clear criteria for high-risk patients. Therefore, this study aimed to clarify the prognosis of high-risk luminal breast cancer based on the data from the MonarchE study criteria. MonarachE investigated the use of abemaciclib as an adjuvant therapy in high-risk luminal breast cancer. There are no reported clinical data on the prognostic value of abemaciclib in high-risk luminal breast cancer. This study examined the clinical data and prognosis of patients eligible for the MonarachE trial.

## Materials and Methods

### Patients

In this study, all subjects were patients who underwent surgery at Tokyo Metropolitan Komagome Hospital from January 2011 to December 2018. Patients were included when it was possible to followup the prognosis of patients who were estrogen-receptor positive, diagnosed with HER2-negative breast cancer with ≥4 metastatic axillary lymph nodes, or with 1-3 metastatic axillary lymph nodes, invasion diameter ≥5 cm, nuclear grade (NG) 3, and Ki67 ≥20%. Compared to the MonarchE phase global trial, our institution uses NG rather than HG, and therefore NG3 was employed in the present study The following inclusion criteria were also employed: patients not receiving endocrine therapy as adjuvant therapy, whereas postoperative radiation therapy and pre- and postoperative chemotherapy and endocrine therapy were allowed. In accordance with the MonarchE study, patients with inflammatory breast cancer, deep vein thrombosis, and occult breast cancer were excluded. For cases treated with chemotherapy, this was considered chemotherapy group if chemotherapy was administered even once, and relative dose intensity (RDI) was not specified. RDI was kept above 80%.

### Pathological information

Pathological diagnosis was made at Tokyo Metropolitan Komagome Hospital by Japanese physicians with specialization in pathology. Pathological evaluation was performed using specimens at the time of surgery; however, in patients who received preoperative chemotherapy, biopsy specimens were used for the examination.

### Staging

For patients not receiving preoperative drug therapy, pathology specimens were used for the final stage. Tumor diameter for patients who received preoperative drug therapy was defined as the largest of the invasive area observed on either magnetic resonance imaging or ultrasonography (US). The number of lymph node metastases was based on computed tomography (CT) and contrast-enhanced if possible, and the number of metastases was defined as the number of clearly enlarged nodes. Lymph nodes were considered metastatic if they were 15 mm or larger. When performed prior to treatment, the diagnosis of positive lymph node metastasis was based on cytological results, and when diagnostic imaging revealed multiple lymph node metastases, they were considered positive even without pathological findings. Even if lymph node metastasis had shrunk after preoperative chemotherapy, sentinel node metastasis was omitted, and axillary lymph node dissection was performed in cases determined to be positive for metastasis before treatment. The results were compared with those of the MonarchE study. In accordance with the MonarchE trial, Cohort 1 was defined as ≥4 lymph node metastases or 1-3 lymph node metastases with a tumor diameter of ≥5 cm, NG 3, or histologic grade III, while Cohort 2 was defined as 1-3 lymph node metastases with Ki67 ≥20%. In Cohort 1, cases with Ki67 ≥20% were defined as “Ki67 high,” and cases with Ki67 <20% were defined as “Ki67 low.”

### Data collection

Patients were selected from our surgical data system, and their clinical information and pathological data were collected from their medical records retrospectively. Image studies and diagnostic reports were obtained from the radiology reporting system.

### Postoperative surveillance

Postoperative breast cancer surveillance at our hospital includes MG. Additional tests such as US blood tests, CT, and PET-CT were performed only in cases deemed necessary by the clinician. When recurrence was suspected, a regenerative examination was performed if necessary. Clinically, if recurrence was judged to be due to imaging studies alone, we did not perform a regenerative examination.

### Statistical analysis according to prognosis

Clinical information was collected retrospectively by analyzing patients’ medical records, and statistical analysis was performed using the EZR software v.1.61 ^(23)^. A *P* value <0.05 was specified as a significant difference, and IDFS, disease-free survival, and OS were calculated using Kaplan-Meier curves.

### Ethical approval and consent to participate

This study adheres to the Declaration of Helsinki standards for conducting scientific research, the Clinical Research Act (Act No. 16 of 2017), the Enforcement Regulations of the Clinical Research Act (Ministry of Health, Labor and Welfare Ordinance No. 17 of 2018), and related notices. Furthermore, this study was conducted using data approved by the Ethics Review Committee of our hospital. Patients were clearly informed that their clinical data would be used for research, and comprehensive consent was obtained to conduct this study.

## Results

### Patients’ characteristics and pathological data

One hundred and fifty-two patients were included in the study, and Table 1 shows their characteristics and pathological staging and factors. Patients’ mean age at first visit was 51.5 years (range: 32-87 years), and the mean observation period was 64.7 months (range; 9.7-119 months). Seventy-five cases (49%) were premenopausal, and 77 (51%) were postmenopausal. Of all the patients included, 127 (84%) were treated with chemotherapy, 47 (31%) with neoadjuvant chemotherapy (NAC), and 80 (53%) with adjuvant chemotherapy. Postoperative endocrine therapy was administered in 150 cases (99%), including tamoxifen in 76 cases (50%), anastrozole in 42 (27%), letrozole in 28 (18%), and exemestane in 4 cases (3%) (Table 1).

**Table 1.** Patients’ Characteristics and Pathological Stage and Factors.

	n = 152	Overall %
Mean age, years, (range)	51.5 (32-87)	
Mean observation period, months, (range)	64.7 (9.7-119.0)	
Menopausal status		
Premenopausal	75	49%
Postmenopausal	77	51%
Chemotherapy		
Yes	127	84%
Neoadjuvant	(47)	(31%)
Postoperative	(80)	(53%)
No	25	16%
Preoperative endocrine therapy	2	1%
Types of chemotherapy		
Anthracyclines and taxane	106	70%
Anthracycline only	3	2%
Taxane only	18	12%
Postoperative endocrine therapy		
Yes	150	99%
(Tamoxifen)	(76)	(50%)
(Anastrozole)	(42)	(27%)
(Letrozole)	(28)	(18%)
(Exemestane)	(4)	3%
No	2	1%
LHRH agonist	15	10%
Stage		
Stage IIA	25	16%
Stage IIB	33	22%
Stage IIIA	66	43%
Stage IIIB	1	1%
Stage IIIC	27	18%
Median invasive diameter, cm (range)	2.8 (0.7-26.5)	
Invasive diameter ≥5 cm	55	36%
Median lymph node metastases (range)	3 (137)	
Four or more lymph node metastases	68	44%
pCR after NAC	3	7%
Nuclear Grade		
1, 2	56	37%
3	96	63%
Ki67		
Ki67 ≧ 20%	39	26%
Ki67 < 20%	90	59%
unknown	24	16%
Progeterone receptor		
Positive	126	83%
Negative	26	17%
Cohort		
1	152	100%
(High)	(39)	(26%)
(Low)	(90)	(59%)
(Unknown)	(36)	(24%)
2	0	

pCR, pathological complete response; NAC, neoadjuvant chemotherapy

More specifically, Stage IIA was in 25 cases (16%), Stage IIB in 33 cases (22%), and Stage IIIA in 66 cases (43%), Stage IIIB in 1 case (1%), and Stage IIIC in 27 cases (18%). A diameter of ≥5cm was found in 55 cases (36%). Lymph nodes metastasis ≥4 was found in 68 cases (44%), NG3 in 96 cases (63%), and Ki67 ≥20% in 39 cases (26%). All cases were from Cohort 1.

### Occurrence of events

**Figure 1.**
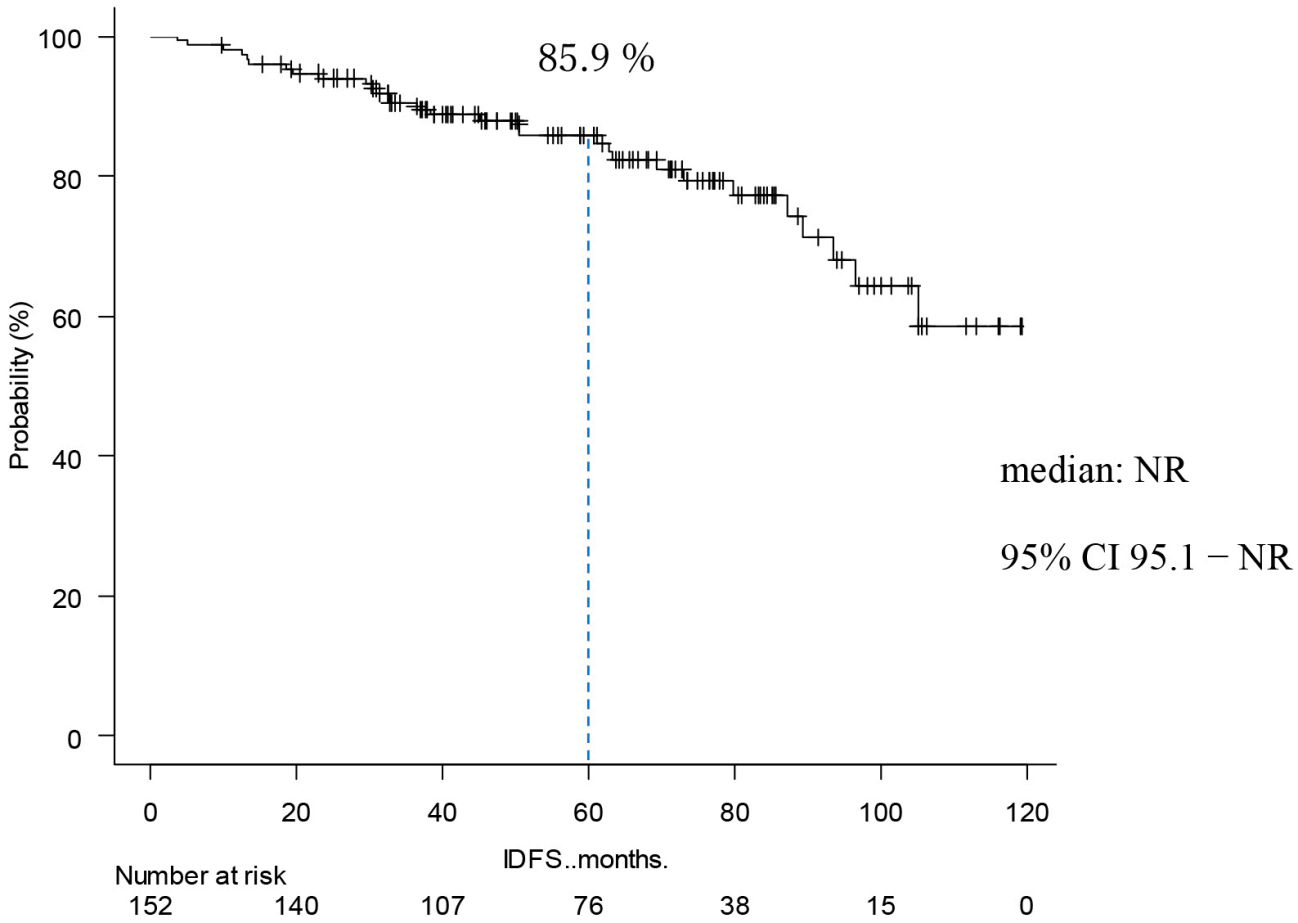
Kaplan-Meier Curve of IDFS The IDFS at 5 years was 89.5%. The median was not reached.

**Figure 2.**
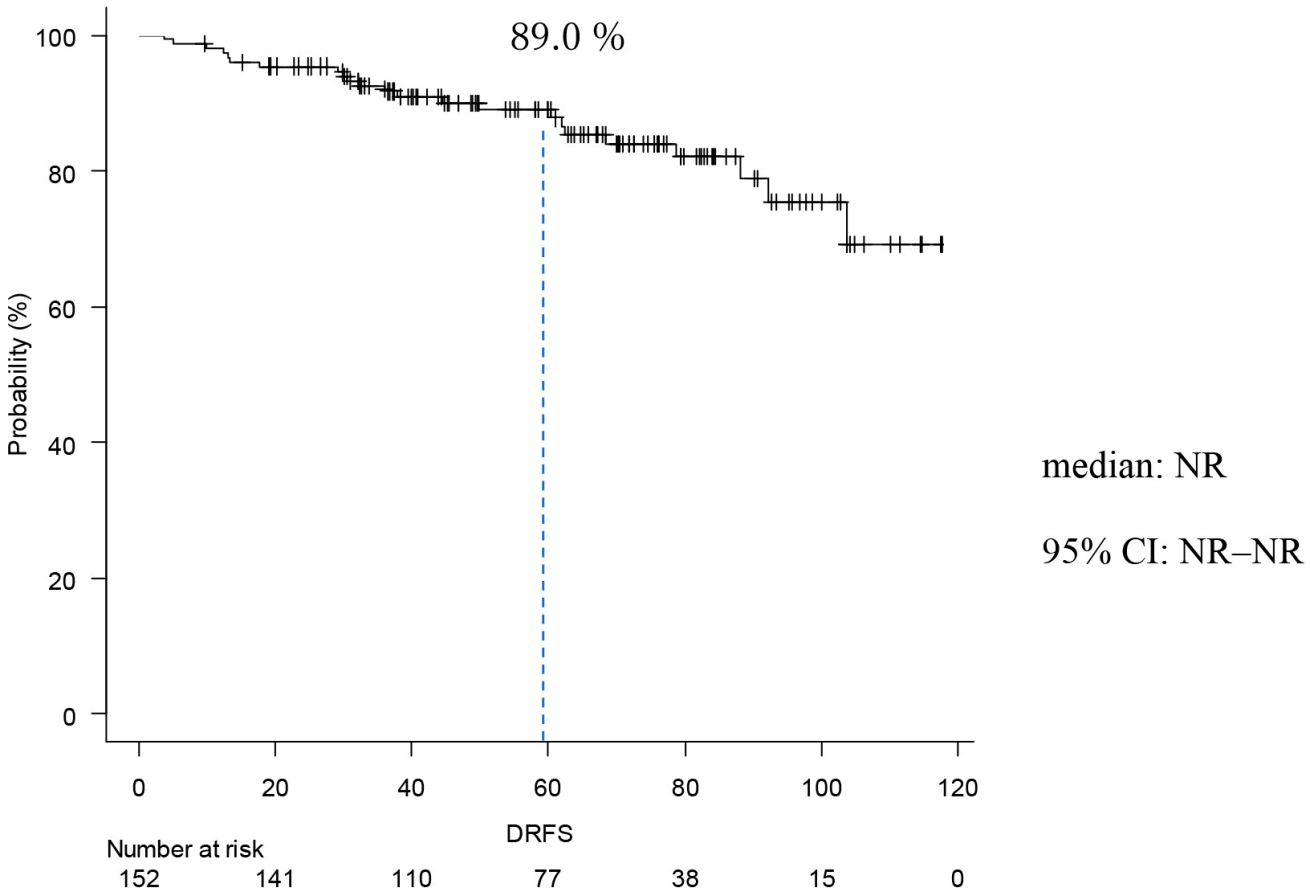
Kaplan-Meier Curve of DRFS The DRFS at 5 years was 89.0%. The median was not reached.

**Figure 3.**
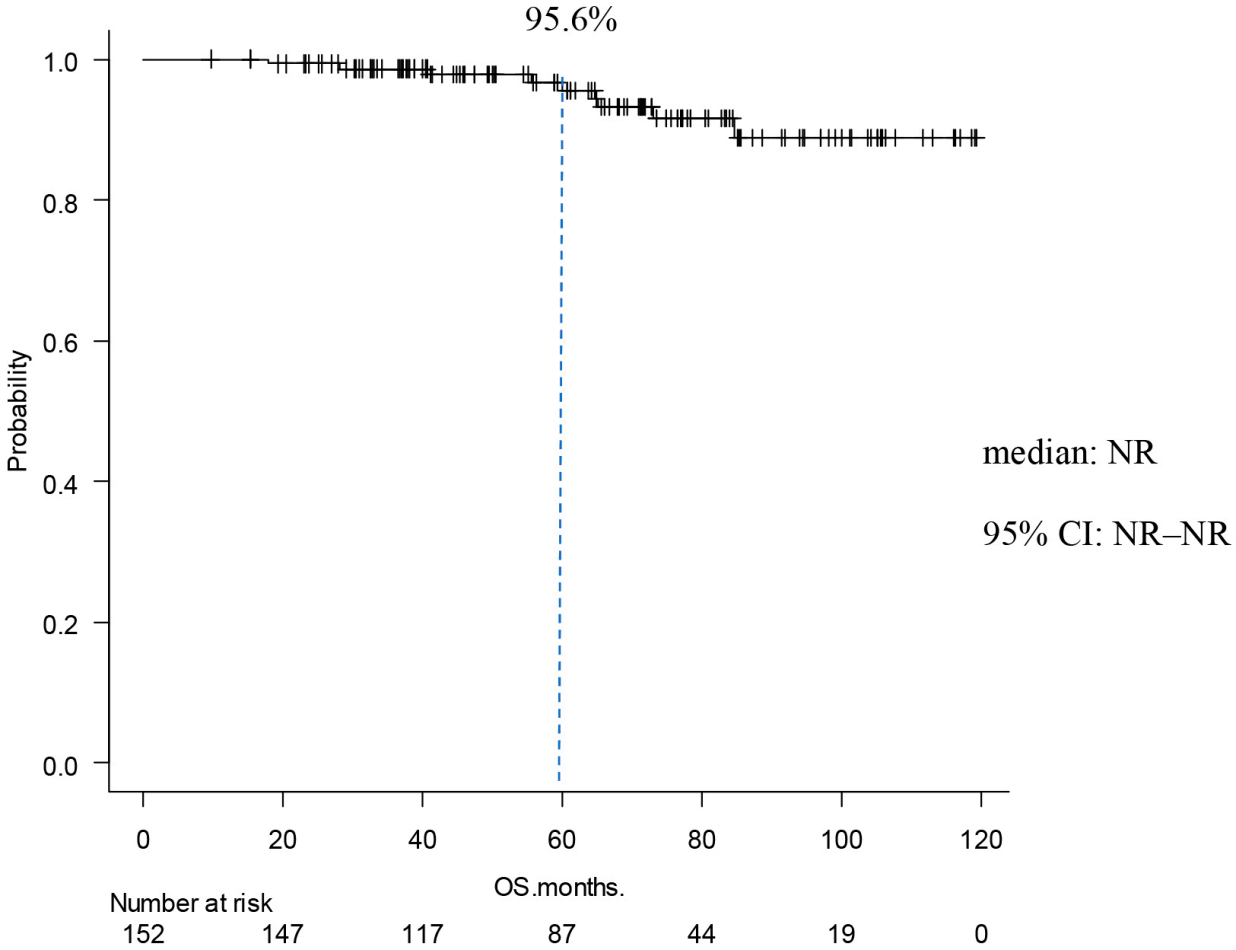
Kaplan-Meier Curve of OS The OS at 5 years was 95.0%. The median was not reached.

**Figure 4.**
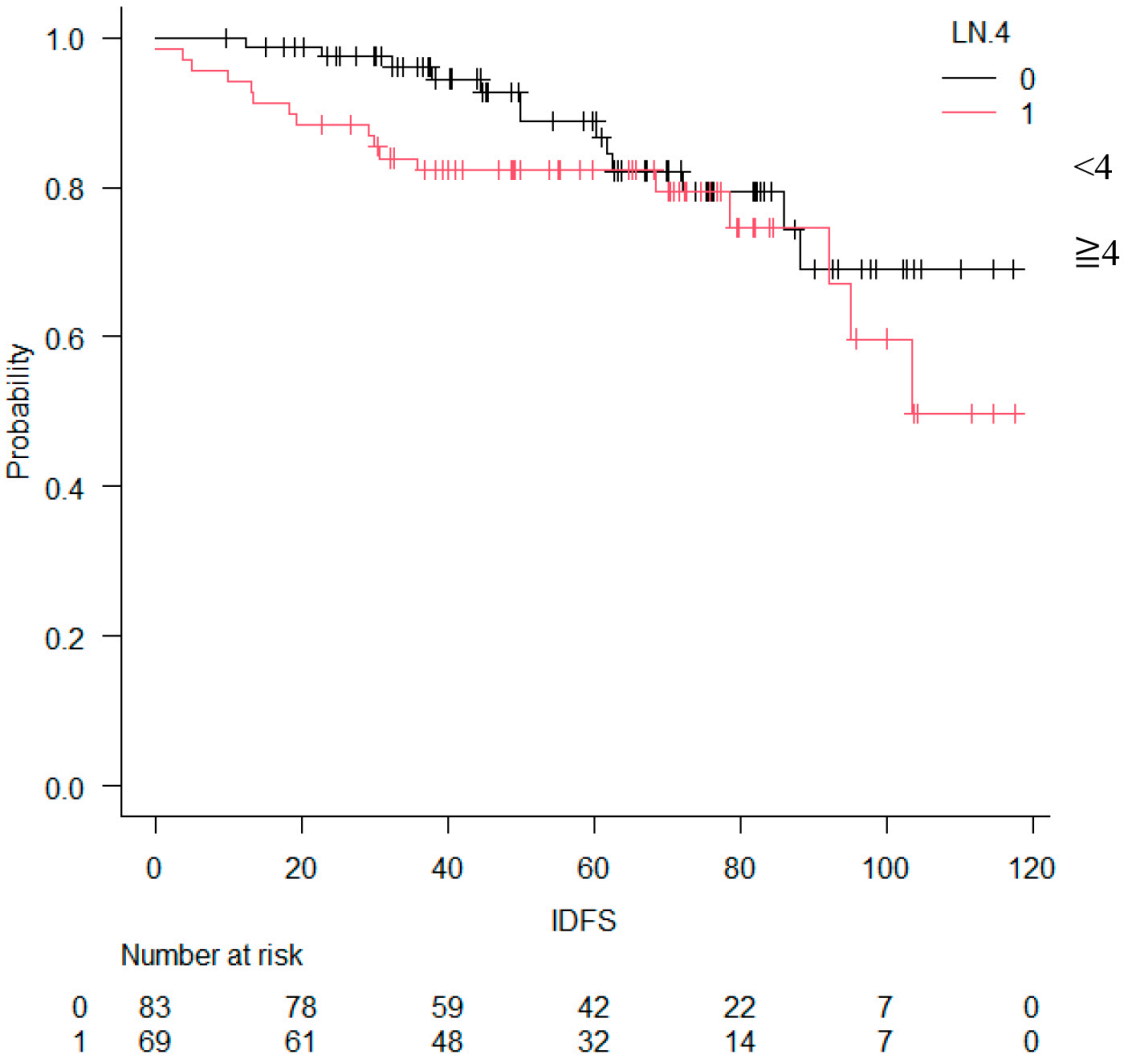
Comparison of four or more lymph node metastases and the following IDFS The Kaplan-Meier curve of IDFS divided by number of lymph node metastases (p = 0.35, HR: 1.42 [95% CI: 0.68-2.96]).(p = 0.225, HR: 1.57 [95% CI: 0.75 3.25]).

Table 2 shows the occurrence of events and prognosis. IDFS events occurred in 30 cases (20%) The reason of IDFS events was invasive disease in 27 cases (18%). Six patients (4%) had local/regional recurrence, 19 patients (13%) had distant recurrence, and 5 (3%) had a second primary neoplasm. Three patients (2%) died. IDFS at 5 years was 85.9 % (Figure 1). DRFS events occurred in 19 patients (13%), and DRFS at 5 years was 89.0% (Figure 2). As for the final outcome, there was survival in 143 cases (91%) and death in 9 cases (6%). Of these deaths, 5 (3%) resulted from breast cancer (Table 2). OS rate at 5 years was 95.6 % (Figure 3). There was no significant difference between the two groups in IDFS for 1-3 lymph nodes versus ≥4 lymph nodes (*p* = 0.225, HR: 1.57; 95% confidence Interval [CI]: 0.75-3.25). Finally, IDFS at 5 years was 88.6% for patients with ≥4 and 83.0% for patients with <4 lymph nodes (Figure 4). Table 3 shows the analysis broken down by the presence or absence of ID events. There were no significant differences in chemotherapy, menstrual status, number of lymph node metastases, NG, tumor diameter, or Ki67 with or without ID events.

**Table 2.** Occurrence Events and Prognosis.

	n =152	%
IDFS Event	30	20%
Patients with invasive disease, first recurrence	27 (18%)	18%
(Local/regional recurrence)	(6)	(4%)
(Distant recurrence)	(19)	(13%)
(Contralateral recurrence)	(0)	
(Second primary neoplasm)	(5)	(3%)
Death from any cause without invasive disease	3	2%
Median time to IDFS event, months（range）	60 (3.8119)	
DRFS event	19	13%
Median time to DRFS event, months（range）	34.7 (3.9105)	
Bone	11	7%
Liver	5	3%
Lung	5	3%
Brain	1	0.7%
Lymph node	3	2%
Pleura	0	
Central nervous system	0	
Soft tissue	0	
Skin	0	
Peritoneum	0	
Other	1	0.7%
Death from any cause without	3	2%
Distant recurrence		
Final Outcomes		
Survival	143	91%
Death	9	6%
(breast cancer)	(5)	(3%)

IDFS, invasive-disease-free survival; DRFS, distant-relapse-free survival

**Table 3.** Occurrence of IDFS Events.

IDFS events	Yes (n = 30)	No (n = 122)	*P* value
Chemotherapy			0.17
Yes	22 (73%)	104 (85%)	
No	8 (27%)	18 (15%)	
Menopausal			
Premenopausal	12 (40%)	63 (52%)	0.31
Postmenopausal	18 (60%)	59 (48%)	
Lymph Node Metastasis			
≤3	13 (43%)	70 (57%)	0.22
>4	17 (57%)	52 (43%)	
NG			
NG1, 2	14 (47%)	42 (34%)	0.29
NG3	16 (53%)	80 (66%)	
Diameter			
<5cm	15 (50%)	40 (33%)	0.09
≧ 5cm	15 (50%)	82 (67%)
Ki-67			
<20%	10 (33%)	29 (24%)	0.22
≧20%	14 (47%)	76 (62%)	
Unknown	6 (20%)	17 (14%)	

IDFS, invasive-disease-free survival; NG, nuclear grade

### Comparison with the MonarchE study

Our findings were then compared with the MonarchE study results. In the MonarchE study, 2,681 cases (95%) received perioperative chemotherapy, a higher percentage of chemotherapy than in current study (Table 4). The number of patients receiving sequential anthracycline and taxane was 106 cases (70%) in this study and 2,349 cases (83.7%) in the MonarchE study. Furthermore, there were 58 (38%) Stage II and 94 (62%) Stage III breast cancer cases. In contrast, the MonarchE study included 712 cases (25.4%) of Stage II and 2,081 cases (74.5%) of Stage III (p = 0.01).

**Table 4.** Comparison with the MonarchE Study.

	Present Study (n = 152)	MonarchE Study (n = 2,808)
Menopausal		
Premenopausal	75 (49%)	1,221 (43.5%)
Postmenopausal	77 (51%)	1,587 (56.5%)
Chemotherapy		
Yes	127 (84%)	2,681 (95.5%)
(Neoadjuvant)	47 (31%)	1,039 (37.0%)
(Postoperative)	80 (53 %)	1,642 (58.5%)
No	25 (16%)	127 (4.5%)
Types of Chemotherapy		
Anthracyclines and taxane	106 (70%)	2,349 (83.7%)
Anthracycline only	3 (2%)	151 (5.3%)
Taxane only	18 (12%)	217 (7.7%)
Postoperative Endocrine Therapy		
Yes	150 (99%)	2,808 (100%)
(Tamoxifen)	76 (50%)	857 (30.7%)
(Anastrozole)	42 (27%)	611 (21.9%)
(Letrozole)	28 (18 %)	1,092 (39.1%)
(Exemestane)	4 (3%)	225 (8.1%)
No	2 (1%)	0
LHRH agonist	15 (10%)	606 (21.7%)
Stage		
Stage ⅠA	0	2 (0.1%)
Stage Ⅱ	58 (38%)	712 (25.4%)
Stage Ⅲ	94 (62%)	2,081 (74.5%)
Invasive diameter 5 cm or more	55 (36%)	610 (21.7%)
Lymph node metastasis ≧ 4	68 (44%)	1,680 (59.8%)
Ki67 ≧ 20%	39 (26%)	953 (34%)
Ki67 < 20%	90 (59%)	1,855 (66%)

### Comparison between premenopausal and postmenopausal cases

**Figure 5.**
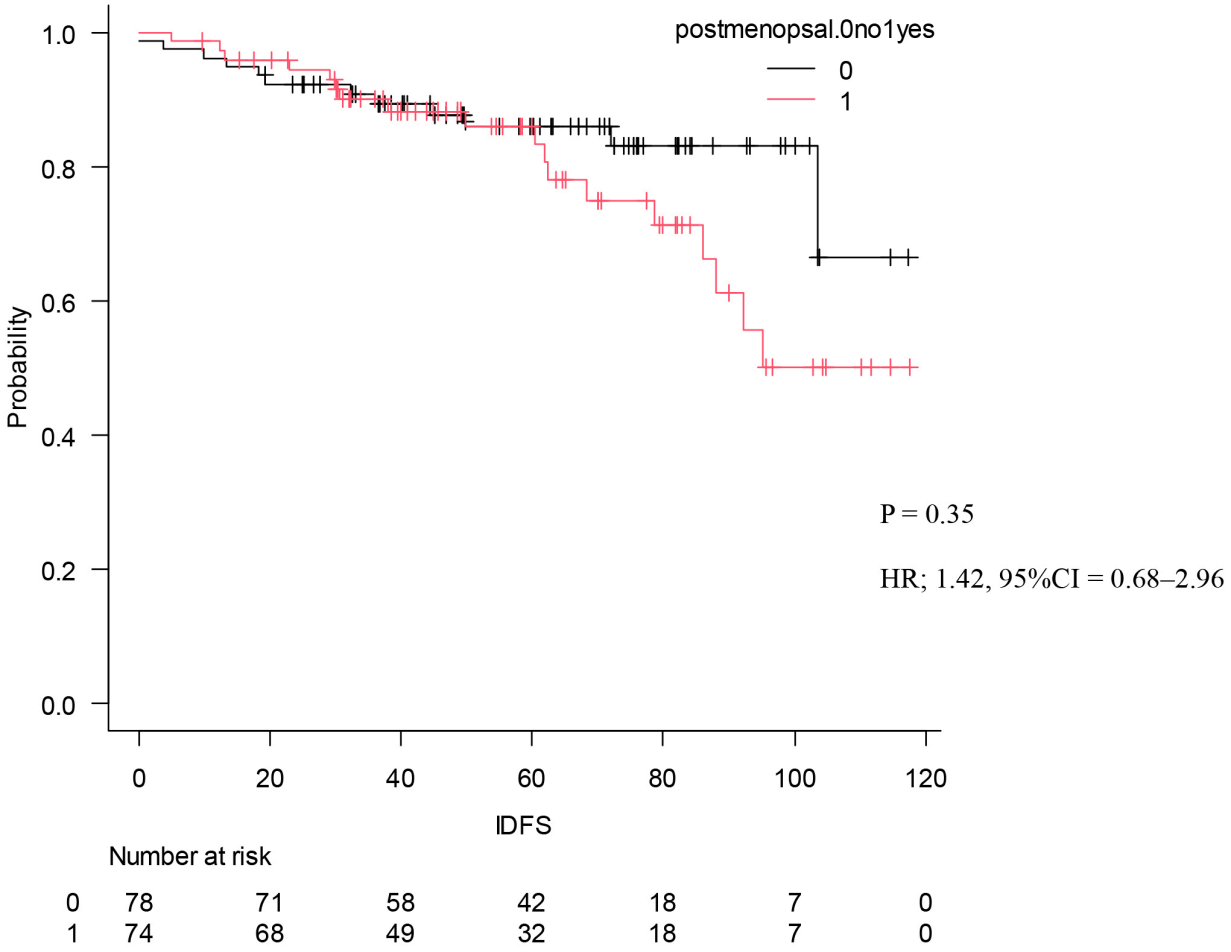
Comparison of premenopausal and postmenopausal the following IDFS The Kaplan-Meier curve of IDFS divided by menopause is shown. (p = 0.35, HR: 1.42 [95% CI: 0.68-2.96]).

Our findings show a higher percentage of patients with premenopausal breast cancer compared to that in the MonarhE study (49% vs. 43.5%) ^(20)^. Although there were no significant differences with respect to stage and pathology, as shown in Table 5, the presence or absence of chemotherapy was significantly different between the patients in post- and premenopausal studies. Seventy patients (93%) were treated with chemotherapy before and 56 (73%) were treated with chemotherapy after menopause, with a clear tendency for more cases to be treated with chemotherapy before menopause (*p* < 0.01). The content of endocrine therapy also tended to be higher for tamoxifen in premenopausal patients (*p* < 0.01). Kaplan-Meier curves were drawn for IDFS to compare the two groups, and no significant difference was found (Figure 5).

**Table 5.** Comparison between Premenopausal and Postmenopausal Patients.

	Premenopausal (n = 75)	Postmenopausal (n = 77)	*P* value
Age	46.1 ± 6.8	65.6 ± 9.7	< 0.01
Stage			
Stage Ⅱ	31 (41%)	27 (35%)	0.51
Stage Ⅲ	44 (59%)	50 (65%)	
Diameter			
<5 cm	45	52	0.40
>5 cm	30	25	
Lymph Node Metastasis			
<3	45 (60%)	38 (49%)	0.20
≥4	30 (40%)	39 (51%)	
NG			
NG1, 2	32 (43%)	24 (31%)	0.18
NG3	43 (57%)	53 (67%)	
Chemotherapy			
Yes	70 (93%)	56 (73%)	<0.01
Neoadjuvant			
Adjuvant			
No	5 (7%)	21 (27%)	
Postoperative Endocrine Therapy			
Yes			Tamoxifen *vs.* Aromataze inhibitor < 0.01
(Tamoxifen)	72 (96%)	4 (5%)	
(Anastrozole)	0	42 (55%)	
(Letrozole)	2 (3%)	26 (34%)	
(Exemestane)	0	4 (5%)	
No	1 (1%)	1 (1%)	
LH-RH agonist	10 (13%)	0	<0.07

NG, nuclear grade

## Discussion

The present study was conducted to investigate the postoperative course of high-risk luminal breast cancer. Chemotherapy was given to 127 cases (84%) Premenopausal was in 75 cases (49%). Stage II was 58 cases (38%), and Stage III was 86 cases (62%). In the MonachE trial, 25.5% were Stage II and 74.5% were Stage III cases ^(20)^. The fact that the percentage of chemotherapy in the Monarch E study was higher than in our study may be attributed to the higher stage group. Patients in Cohort 1 had high-risk features defined as either ≥4 positive axillary lymph nodes (pALN), or 1-3 pALN and Grade 3 disease and/or tumor ≥5 cm. Cohort 2, included patients with 1-3 pALN, Grade <3, tumor <5 cm, and Ki-67 ≥20%, who started enrollment later; thus the data remain immature in this study, and all applicable cases were from Cohort 1. This can be explained considering the fact that the present study used NG rather than HG and also because of the missing Ki67 values in some cases. Age-specific incidence rates of breast cancer in East Asian countries have been reported to differ from those in Western countries. Thus, the peak breast cancer incidence rate for women in Japan, Taiwan, and South Korea is 40s, whereas that for Western women is 70s ^(24), (25), (26), (27)^. In this study, the rate of premenopausal breast cancer tended to be higher than in the MonarchE study. In addition, a higher percentage of patients with premenopausal breast cancer received chemotherapy. This may be because patients with postmenopausal breast cancer are more likely to be elderly, thereby rendering it more difficult to administer chemotherapy, but also because these patients have a worse prognosis compared to postmenopausal ones. Although premenopausal breast cancer was expected to have a worse prognosis ^(28)^, IDFS was not significantly different between premenopausal and postmenopausal patients. This may be because a higher proportion of patients with premenopausal breast cancer are receiving chemotherapy. In addition, tamoxifen was used in most cases of premenopausal breast cancer, which might be attributed to Japanese insurance policies not allowing the concomitant use of LH-RH agonist and aromatase inhibitors. Furthermore, the number of patients treated with LH-RH agonist before menopause was smaller than expected, which might be partly because menopause is caused by chemotherapy. IDFS at 5 years was 85.0%, DRFS at 5 years was 89.0%, and OS at 5 years was 95.0%. With the addition of abemaciclib to postoperative hormone therapy for high-risk luminal breast cancer, the MonarchE trial reported improved IDFS and DRFS ^(20)^. In the MonarchE study, IDFS after 5 years was 83.6% in the abemaciclib addition group and 76% in the ET group, indicating the addition effect of abemaciclib. In this study, the IDFS after 5 years was 85%. We also examined DRFS, the secondary endpoint of the monarch E study. In MonarchE study, DRFS after 5 years was 86.0% in the abemaciclib addition group and 79.2 % in the ET group, indicating the addition effect lower than that stated in the MonarchE study abemaciclib, which was lower than that found in the present study and could be explained by the higher number of breast cancers at Stage II or below compared to the MonarchE study. In addition, this may be the reason why our IDFS and DRFS were higher. The number of lymph node metastases based on CT was lower than those stated in the MonarchE study. The OS at 5 years in the current study was 95.6%. In the MonarchE study, neither the abemaciclib nor the ET group reached the median value. It was previously reported that the recurrence rate tends to increase even after 5 years of hormone therapy ^(12)^. Therefore, it was deemed necessary to monitor patients’ progress to confirm whether the addition of perioperative abemaciclib was effective in controlling late recurrence. This study had more cases with lower stages than the MonarchE study. This may explain why IDFS, DRFS, and OS tended to be better than those in the MonarchE study.

Two years of abemaciclib is a physically and financially taxing treatment. As we observed many patients who did not have recurrence even though the MonarchE trial was applicable, it is necessary to consider in the future, which patients really need abemasiclib for 2 years postoperatively.

There are several limitations to this study. First, this was a single-center, backward-looking study. The study was conducted at a single institution for a limited period and was also limited in the number of participants. Second, there are cases with low volume in the chemotherapy because the RDI was not specified group. The reason for not specifying RDI was to show real clinical data. Third, many patients had been treated before Onctotype DX was covered by insurance in Japan. The results of Onctotype DX therapy may have led to the addition or omission of chemotherapy, or to changes in the treatment plan. Late recurrence (i.e., >10 years after surgery) is more common in luminal breast cancer than in other subtype ^(29)^. However, in our study, the median observation period was 0 months, which is not an adequate follow up for HER2-negative breast cancer treated with preoperative chemotherapy. Residual tumor size has been associated with OS, while high Ki67 has been linked to OS and DRFS, according to some reports ^(30)^. Although the number of pathological complete response (pCR) cases was too low to examine, it is necessary to investigate whether the addition of abemaciclib changes the prognosis in non-pCR and pCR cases as a future research topic.

Currently in Japan, additional agents besides chemotherapy and hormonal therapy that can be used in the perioperative treatment of high-risk luminal breast cancer include abemaciclib, olaparib, and S-1. Although the CreateX trial has demonstrated an additional effect of Capecitabine in patients with non-pCR after NAC ^(31)^, CreateX is not covered by insurance in Japan. Some of the indicated cases are covered, and no conclusion has yet been reached as to which option is superior. The patients’ quality of life may be worsened in terms of cost and adverse events, and informed consent is necessary to determine which drug to choose. However, in terms of the largest number of cases, MonarchE study in terms of the largest number of cases. Yet, in reality, many cases do not recur without abemaciclib. Moreover, narrowing down the number of cases that need to be treated would reduce the burden on the patient. Luminal breast cancer has a high incidence of late recurrence, and longer follow up is likely to be necessary in the MonarchE trial and in this study.

### Conclusions

This study examined real-world data of high-risk luminal breast cancer from the MonarchE trial. The proportion of Stage II patients was higher than that in the MonarchE study, the number of patients who received chemotherapy tended to be lower, and the IDFS has been improved.

## Article Information

### Conflicts of Interest

None

### Author Contributions

All authors contributed to this study’s conception and design. Conceptualization and investigation, M.A.; methodology S.M., K.H., Y.K. and G.O.; supervision T.I. and T.A. All authors have read and agreed to the published version of the manuscript

### Approval by Institutional Review Board (IRB)

This study was conducted in accordance with the Declaration of Helsinki and approved by the Institutional Review Board of Tokyo Metropolitan Cancer and Infectious Disease Center Komagome Hospital (protocol no.: 2,971; date: October 11, 2022).
